# Diagnostic and pre-treatment intervals among patients with cervical cancer attending care at the Uganda Cancer Institute: a cross-sectional study

**DOI:** 10.1186/s12905-023-02785-3

**Published:** 2023-11-27

**Authors:** Jackie Lalam Lacika, Henry Wabinga, Joseph Kagaayi, Ronald Opito, Christopher Garimoi Orach, Amos Deogratius Mwaka

**Affiliations:** 1 Department of Community Health & Behavioural Sciences, School of Public Health, College of Health Sciences, Makerere University, P.O Box 7072, Kampala, Uganda; 2 Department of Pathology, School of Biomedical Sciences, College of Health Sciences, Makerere University, P.O Box 7072, Kampala, Uganda; 3 Department of Public Health, School of Health Sciences, Soroti University, P.O Box 211, Soroti, Uganda; 4 Department of Medicine, School of Medicine, College of Health Sciences, Makerere University, P.O Box 7072, Kampala, Uganda; 5 Department of Medicine, Faculty of Medicine, Gulu University, P.O Box 166, Gulu, Uganda

**Keywords:** Cervical cancer, Diagnostic intervals, Pre-treatment intervals, Advanced stage

## Abstract

**Background:**

Majority of patients with cervical cancer in the low- and middle-income countries experience long diagnostic and pre-treatment intervals. This study sought to determine the factors associated with the diagnostic and pre-treatment intervals among patients with cervical cancer.

**Methods:**

This was a cross-sectional study conducted at the Uganda Cancer Institute (UCI) during October 2019 to January 2020. Patients aged ≥ 18 years with histological diagnosis of cervical cancer were consecutively sampled. Data were collected using a pre-tested semi-structured questionnaire and a data abstraction form. Diagnostic intervals, defined as the time between first visit of a patient to a primary healthcare provider to time of getting confirmed diagnosis, of ≤ 3 months was defined as early & >3 months as late. Pre-treatment intervals, which is the time from histological diagnosis to starting cancer chemo-radiotherapy of ≤ 1 month was defined as early and > 1 month as late. Data were analysed using STATA version 14.0. We used modified Poisson regression models with robust variance to determine socio-demographic and clinical factors associated with the intervals.

**Results:**

The mean age of the participants was 50.0 ± 11.7 years. The median diagnostic and pre-treatment intervals were 3.1 (IQR: 1.4–8.2) months and 2.4 (IQR: 1.2–4.1) months respectively. Half of the participants, 49.6% (200/403) were diagnosed early; one in 5 patients, 20.1% (81/403) promptly (within one month) initiated cancer chemo-radiotherapy. Participants more likely to be diagnosed early included those referred from district hospitals (level 5) (aPR = 2.29; 95%CI: 1.60–3.26) and with squamous cell carcinomas (aPR = 1.55; 95%CI: 1.07–2.23). Participants more likely to be diagnosed late included those who first discussed their symptoms with relatives, (aPR = 0.77; 95%CI: (0.60–0.98), had > 2 pre-referral visits (aPR = 0.75; 95%CI (0.61–0.92), and had advanced stage (stages 3 or 4) (aPR = 0.68; 95%CI: 0.55–0.85**)**. Participants more likely to initiate cancer chemo-radiotherapy early included older patients (≥ 60 years) (aPR = 2.44; 95%CI: 1.18–5.03). Patients likely to start treatment late were those who had ≥2 pre-referral visits  (aPR = 0.63; 95%CI: 0.41–0.98) and those that took 3 - 6 months with symptoms before seeking healthcare (aPR = 0.52;95%CI: 0.29 - 0.95).

**Conclusion:**

Interventions to promote prompt health-seeking and early diagnosis of cervical cancer need to target primary healthcare facilities and aim to enhance capacity of primary healthcare professionals to promptly initiate diagnostic investigations. Patients aged < 60 years require targeted interventions to promote prompt initiation of chemo-radiation therapy.

**Supplementary Information:**

The online version contains supplementary material available at 10.1186/s12905-023-02785-3.

## Background

Cervical cancer is the fourth most frequent cancer diagnosed among females worldwide, with an estimated 604,000 new cases and 342,000 deaths in 2020. About 90% of all the new cases and deaths from cervical cancer in 2020 occurred in the low- and middle-income countries (LMICs), mainly in South Eastern Asia and sub Saharan Africa (SSA) [1]. In SSA, the highest regional incidence and mortality rates were seen in Southern & Eastern Africa [1, 2]. In Uganda, 6,959 women were diagnosed with cervical cancer in 2020, and 4,607 women died of the disease [1, 3]. In the LMICs, a high proportion, (65–85%) of women are diagnosed at advanced stages of the disease, and experience low survival [4, 5]. Cancer stage at diagnosis is one of the key factors that influence cervical cancer survival [6–10]. In sub Saharan Africa, women with advanced stages (3/4) had poor relative survival of 20.5% compared to 50.3% for patients with early stage (1/2) [4]. The survival probabilities greatly improve when patients with cervical cancer are timely diagnosed when cancer is at an early stage, and cancer specific treatments started promptly [11, 12]. Down-staging cancer at diagnosis is partly dependent on timely presentation to primary healthcare facilities and diagnosis, timely access to specialist care, and prompt initiation of cancer specific treatment, especially for breast, colorectal, head and neck, and testicular cancers [13–15]. Timely diagnosis and prompt treatment are among the key strategies for combating the high mortality rates of cancer [16]. In addition, timely presentation for healthcare enables early detection of pre-malignant lesions or invasive cancer when treatment is still beneficial [17–19].

The interval from first patient visit to a primary healthcare provider to when he or she gets a confirmed diagnosis of cancer is referred to as the diagnostic interval. On the other hand, the pre-treatment interval is the time from histology cancer diagnosis to the start of cancer specific treatment e.g. chemo-radiotherapy [20]. Cancer patients with longer diagnostic intervals have more advanced stage cancers at diagnosis, and experience poorer outcomes including lower survival [21]. For example, in Ethiopia, a study that involved 212 cervical cancer patients diagnosed between January 2017 and June 2018, showed that 61% of the patients were diagnosed in advanced stage. The patients with long diagnostic intervals of greater than 3 months (adjusted prevalence ratio = 1.31; 95%CI: 1.04–1.71) and those who visited primary healthcare facilities > 3 times before confirmation of cancer diagnoses (adjusted PR = 1.24; 95%CI: 1.07–1.51) were statistically significantly more likely to be diagnosed with advanced stage cancer [22]. In Uganda, there is limited data on the diagnostic & pre-treatment intervals that lead to the best chances of survival for cervical cancer. In this study, we sought to determine the diagnostic and pre-treatment intervals, as well as factors associated with these intervals among cervical cancer patients receiving care at the national specialised cancer treatment facility.

## Methods

### Study design and site

This was a cross-sectional study conducted at the Uganda Cancer Institute (UCI). The UCI is the only public tertiary specialized cancer care centre that provides cancer specific and supportive treatments, and cancer research and training in Uganda [23]. The Institute was started in 1967 as a collaborative research centre between Makerere University, Mulago National Referral hospital, and the USA National Cancer Institute [24, 25]. The UCI has evolved into the oncology centre of excellence for the East African region, providing care to patients with all types of cancers from Uganda, Kenya, Tanzania, Rwanda, South Sudan and the Democratic Republic of Congo (DRC).

### Study population and sampling procedure

Adult (aged ≥ 18years) patients with histology diagnosis of cervical cancer attending care at the institute during the study period were consecutively included in the study. We excluded cervical cancer patients diagnosed through screening, very ill patients, and patients with incomplete medical records e.g. lacking referral forms and histology reports. We limited recruitment to patients diagnosed within 24 months from date of enrolment; this was to allow for reasonable recall of dates of key events including dates of onset of symptoms and first health-seeking, and to minimize selection bias resulting from survivorship effect. We also excluded cervical cancer patients from other countries; this was to account for variation in outcome measures that would result from country context specific influences on the events and factors in the pathways to treatment.

Recruitment was conducted by three female research assistants (RAs) who were trained on the study purpose, objectives, and procedures including consent process, and data collection and storage. Two of the RAs were non-healthcare professional graduates with vast experiences in research data collection. These two RAs did not work at the UCI, and were therefore not expected to influence patients’ responses and decisions to participate in the study. The third research assistant was a nursing officer from the UCI, and her main roles were to identify and retrieve patients’ files for purposes of data abstraction. The study team approached cervical cancer patients who were waiting for consultations and treatments at the out-patients clinic. All admitted stable cervical cancer patients were also approached and requested to participate in the study.

### Data collection

Data collection was conducted between October 2019 and March 2020. Data were collected using a pre-tested (10 patients included in the pre-test), semi-structured questionnaire. The questions were adapted from the African Women Awareness of Breast and Cervical Cancer (AWACAN) tool and the Model of Pathways to Treatment (MPT) [26, 27]. The questionnaire was refined on the basis of data from the pre-test study. Patients who participated in the pre-test were not enrolled in the main study. The refined version of the questionnaire was loaded in the KoBo toolbox software version 2.0 installed in android phones, and administered by the research assistants (Supplementary file 1). The questionnaire included information on: socio-demographic profile of participants, awareness of cervical cancer risk factors and symptoms, cancer symptoms experienced, health-seeking, and dates of key events on the pathway to treatment. The tool also had questions on health system factors including number of times patients visited health facilities before referral to the UCI, distance from patient’s home to nearest health facility, distance from home to the UCI, and disease factors including histology type, tumour grades and cancer stage at diagnoses.

Each research assistant (RA) conducted interview with a participant in a quiet room; non-participants were not allowed in the interview rooms. Each interview lasted about 40 to 60 min. The RAs helped participants to recall dates of key events during data collection by use of the calendar landmark approach, i.e. providing prompts based on salient public events including Presidential election months, Christmas day, and the Independence Day [28]. Data were collected on the clinic days and on every day on the wards, until the sample size was achieved. Following the face-to-face interviews, the nurse RA helped to retrieve patients’ files (case notes) from the records department for data abstraction by linking the participants’ study numbers with the patients’ file numbers. The RAs abstracted data including on patients’ date of histology diagnosis, date of referral to UCI, cancer stage at diagnosis, and date of start of cancer specific treatments. JLL supervised the data collection process. At the end of every day of data collection, JLL ensured that the RAs downloaded data from their android phones onto the investigator’s laptop secured with a password to promote confidentiality of data. In order to promote completeness, consistency and accuracy of data before storage, JLL reviewed data with RAs, ensured that the patients’ file numbers and study numbers were appropriately matched, and then checked contents for completeness. Questionnaires with incomplete data were filled by revisiting the patients and or the patients’ files. Patients who were recruited from the outpatient were reached through phone calls or during their next scheduled clinic visits.

### Data management and analysis

Data downloaded from Kobo toolbox 2.0 software were exported to Excel 2013. The biostatistician (RO) and JLL conducted data cleaning and editing. RO exported data from excel to STATA version14.0 for further cleaning, coding and analysis. There were two primary study outcomes; diagnostic interval (DI) obtained by subtracting date of first consultation with the primary healthcare professionals (PHP) from the date of histology diagnosis of cervical cancer, and pre-treatment interval (PTI) obtained by subtracting date of histological diagnosis of cervical cancer from the date of start of cancer chemo-radiotherapy at the UCI. Proportions were used to describe categorical variables, while continuous variables were summarized using medians with their respective interquartile ranges, and mean with their respective standard deviations. Associations between independent variables including socio-demographic characteristics and health system factors with the primary study outcomes were established using Chi-square tests. The outcome variables were dichotomized; diagnostic interval < 3 months and ≥ 3months, and pre-treatment intervals ≤ 1 month and > 1 month. Participants who had a diagnostic interval less or equal to 3 months (90 days) were considered as diagnosed early. And participants who had a pre-treatment interval of less or equal to one month (30 days) were considered to have started cancer specific treatment promptly/early. The cut-off point for diagnostic interval (3 months), the time period which has survival benefit is based on data from breast cancer observational studies which showed that diagnosis of symptomatic breast cancer before 3 months, compared to delays of 3–6 or more months lead to poorer survival [13]. Similarly, it has been shown that the optimum pre-treatment interval for starting breast cancer specific treatment is 1–2 months [29–31]. Among cervical cancer patients, a pre-treatment cut off time of three months (90 days) was evaluated; the study showed that cervical cancer patients who started cancer specific treatments after 90 days or 180 days had poorer overall survival compared to those who started cancer specific treatments within 90 days of diagnosis [12]. However, in this study, start of cancer specific treatment (i.e. chemo-radiotherapy) within one month was considered the cut off for timely initiation of cancer specific therapies including chemotherapy and or radiotherapy (chemo-radiotherapy). Modified Poisson Regressions with robust variance was used during multivariable analyses to determine the magnitude of associations and factors associated with the diagnostic and pre-treatment intervals. Inclusion of variables into the multivariable regression models was based on clinical relevance and research question, rather than a predetermined p-value during bivariate analyses. In the regression models, outputs were defined as follows: diagnostic interval less than 3 months (outcome of interest) was denoted with “Yes” (= 1) and “No” (= 0); and for the pre-treatment interval, an interval less than one month (outcome of interest) was denoted with “Yes” (= 1) and “No” (= 0). Consequently, adjusted prevalence ratios (aPR) > 1 denotes early diagnosis and prompt onset (within < 1 month) of adjuvant chemo-radiotherapy. We report prevalence ratios with their corresponding 95% confidence intervals as measures of effect sizes. Variables with two-sided p-value < 0.05 were considered statistically significant.

## Results

### Socio-demographic & clinical characteristics of participants

A total of 403 participants had complete data & were included in the analysis; their mean age was 50.0 ± 11.7years. Most participants (80.6%; n = 325) had 3 or more children. Majority of the participants had early stage cancer (stage I [11.2%, n = 45], and stage II [48.9%, n = 197]) at diagnosis, and squamous cell carcinoma (86.3%, n = 348) was the predominant histological subtype (Table 1).

**Table 1:** Socio-demographic characteristics of participants

Characteristics	Number (N)	Percentage (%)
**Age group (Years)**		
< 40	79	19.6
40–49	130	32.3
50–59	108	26.8
≥ 60	86	21.3
**Marital status**		
Single	20	5.0
Divorced	59	14.6
Married	200	49.6
Widowed	124	30.8
**Residence**		
Rural	225	55.8
Urban	178	44.2
**Education**		
No formal education	86	21.3
Primary education	149	37.0
Secondary education	125	31.0
Tertiary education	43	10.7
**Formal employment Status**		
Unemployed	287	71.2
Employed	116	28.8
**Region of country patient comes from**		
Central	177	43.9
Eastern	95	23.6
Northern	39	9.7
Western	92	22.8
**Family History of Cervical Cancer**		
Yes	31	7.7
No/Don’t Know	372	92.3
**Prior Information on Cervical Cancer**		
Yes	339	84.1
No	64	15.9
**Parity (Number of biological children)**		
≤ 3	78	19.4
> 3	325	80.6
**Knowledge of Pap Smear**		
Yes	374	92.8
No	29	7.2
**Symptoms first discussed with**		
Health workers	72	17.9
Relative	298	73.9
Spiritual/Traditional healer	33	8.2
**Cancer stage at diagnosis**		
I	45	11.2
II	197	48.9
III	154	38.2
IV	7	1.7
**Histology type**		
Squamous cell carcinoma	348	86.3
Adenocarcinoma/others	55	13.7

### Diagnostic & pre-treatment intervals

The median time from first clinical visit to histology diagnosis (diagnostic interval) was 3.1 months (IQR: 1.4–8.2); and the median time from histology diagnosis to first cycle of cancer specific treatment (either chemotherapy or chemo-radiotherapy), the pre-treatment interval, was 2.4 months (IQR: 1.2–4.1) (Table 2).

**Table 2:** Diagnostic and pre-treatment intervals

Interval	Description	Median (Months)	Interquartile Range (IQR)
Diagnostic Interval (DI)	Time from first health facility visit to histology diagnosis	3.1	1.4–8.2
Pre-treatment Interval1 (PTI)	Time from Biopsy diagnosis to first cycle of cancer specific treatment	2.4	1.2–4.1

### Association of diagnostic intervals with patients’ socio-demographic, clinical and health system factors

Half of participants (50.4%) were diagnosed after three or more months from first visit to primary healthcare facilities. Participants who first discussed their symptoms with relatives were less likely to be diagnosed early, adjusted prevalence ratio (aPR) = 0.77 (95%CI, 0.60–0.98) as compared to those who discussed symptoms with the health workers. Participants referred to UCI from the district and regional referral hospitals had two-fold higher prevalence of early diagnosis compared to those who were self-referred, aPR = 2.29 (95%CI, 1.60–3.26). Participants who visited the referring sites more than twice before referral were 25% less likely to be diagnosed early compared to those who visited the sites for 2 or fewer times, aPR = 0.75 (95%CI, 0.61–0.92). And participants with stage III/IV cervical cancer were 32% less likely to be diagnosed early compared to those in stages I/II, aPR = 0.68 (95%CI, 0.55–0.85) (Table 3).

**Table 3:** Factors associated with the diagnostic intervals

Factor	Early Diagnosis (DI ≤ 3months) n (%)	Late Diagnosis (DI > 3months) n (%)	Unadjusted Prevalence Ratio (95%CI)	Adjusted Prevalence Ratio (95%CI)
**Age/years**				
< 40	44 (55.7)	35 (44.3)	1.00	1.00
40-<50	65 (50.0)	65 (50.0)	0.89 (0.69–1.17)	0.92 (0.70–1.21)
50-<60	47 (43.5)	61 (56.5)	0.78 (0.58–1.05)	0.75 (0.55–1.02)
≥ 60	44 (51.2)	42 (48.8)	0.92 (0.69–1.22)	1.06 (0.76–1.47)
**Marital Status**				
Single	11 (55.0)	9 (45.0)	1.00	1.00
Divorced	28 (47.5)	31 (52.5)	0.86 (0.53–1.39)	0.85 (0.52–1.36)
Married	100 (50.0)	100 (50.0)	0.91 (0.60–1.38)	0.94 (0.60–1.47)
Widowed	61 (49.2)	63 (50.8)	0.89 ( 0.58–1.38)	0.83 (0.53–1.30)
**Residence**				
Rural	105 (46.7)	120 (53.3)	1.00	1.00
Urban	95 (53.4)	83 (46.6)	1.14 (0.94–1.39)	1.14 (0.92–1.40)
**Education**				
None	40 (46.5)	46 (53.5)	1.00	1.00
Primary	72 (48.3)	77 (51.7)	1.04 (0.78–1.40)	1.04 (0.79–1.37)
Secondary	65 (52.0)	60 (48.0)	1.12 (0.84–1.48)	1.07 (0.78–1.47)
Tertiary	23(53.5)	20(46.5)	1.15 (0.80–1.65)	1.08 (0.72–1.61)
**Formal employment Status**				
Unemployed	132 (46.0)	155 (54.0)	1.00	1.00
Employed	68 (58.6)	48 (41.4)	1.28 (1.05–1.55)	1.22 (0.96–1.55)
**Prior Information on Cervical cancer**				
No	28 (43.8)	36 (56.2)	1.00	1.00
Yes	172 (50.7)	167 (49.3)	1.16 (0.86–1.56)	1.07 (0.80–1.43)
**Family History of Cervical cancer**				
No/Don’t know	185 (49.7)	187 (50.3)	1.00	1.00
Yes	15 (48.4)	16 (51.6)	0.97 (0.67–1.42)	0.98 (0.69–1.39)
**Ever done PAP Smear**				
No	42 (58.3)	30 (41.7)	1.00	1.00
Yes	141 (47.3)	157 (52.7)	1.33 (0.83–2.15)	1.39 (0.91–2.12)
**Person first Discussed symptoms with**				
Health workers	42 (58.3)	30 (41.7)	1.00	1.00
Relative	141 (47.3)	157 (52.7)	0.81 (0.65–1.02)	0.77 (0.60–0.98)*
Spiritual or Traditional healer	17 (51.5)	16 (48.5)	0.88 (0.60–1.29)	1.00 (0.68–1.49)
**Level of Referring health facility (HF)**				
Self-referral	24 (34.3)	46 (65.7)	1.00	1.00
Private Clinics	10 (40.0)	15 (60.0)	1.17 (0.65–2.08))	1.35 (0.78–2.34)
Level4 (HCIVs)	13 (61.9)	8 (38.1)	1.81 (1.13–2.88)	1.54 (0.97–2.45)
Level5 (DHs)	40 (76.9)	12 (23.1)	2.24 (1.56–3.21)	2.29 (1.60–3.26)*
Level6 (RRHs)	67 (46.8)	76 (53.2)	1.37 (0.95–1.98)	1.40 (0.99-2.00)
Level7 (NRHs)	46 (50.0)	46 (50.0)	1.46 (0.99–2.14)	1.36 (0.93–1.97)
**Distance to Nearest HF**				
< 5 km	138 (54.5)	115 (45.5)	1.00	1.00
≥ 5 km	62 (41.3)	88 (58.7)	0.76 (0.61–0.95)	0.84 (0.68–1.04)
**No of Pre-referral Visits**				
≤ 2 times	53 (60.9)	34 (39.1)	1.00	1.00
> 2times	147 (46.5)	169 (53.5)	0.76 (0.62–0.94)	0.75 (0.61–0.92)*
**Composite stage**				
I/II	134 (55.4)	108 (44.6)	1.00	1.00
III/IV	66 (41.0)	95 (59.0)	0.74 (0.59–0.92)	0.68 (0.55–0.85)*
**Histology type**				
Adenocarcinoma and others	19 (34.5)	36 (65.5)	1.00	1.00
Squamous cell carcinoma	181 (52.0)	167 (48.0)	1.51 (1.03–2.20)	1.55 (1.07–2.23)*

HF = health facility

Bold *=Significant at multivariate level with P < 0.05. All factors in the table were adjusted for each other

### Association of pre-treatment intervals with patients’ socio-demographic, clinical and health system factors

Majority of participants (79.9%; n = 322) started cancer specific treatment after 1 month of reporting to the UCI. Patients aged 60 years and above were two and half times more likely to initiate cancer specific treatments within first month of reporting to the treatment centre compared to the younger patients (aged < 40 years), aPR = 2.44; (95%CI: 1.18–5.03). On the other hand, participants who visited the primary healthcare facilities more than twice before referral for cancer diagnosis and/or treatment were 37% less likely to be initiated on treatment early as compared to those who were referred within the first two visits, aPR = 0.63(95%CI, 0.41–0.98), (Table 4).

**Table 4:** Factors associated with pre-treatment intervals:

Factor	Early Treatment (PTI ≤ 1 month) n = 81 (20.1%)	Late Treatment (PTI > 1 month) n = 322 (79.9%)	Unadjusted Prevalence Ratio (95%CI)	Adjusted Prevalence Ratio (95%CI)
**Age/years**				
< 40	11 (13.9)	68 (86.1)	1.00	1.00
40-<50	28 (21.5)	102 (78.5)	1.54 (0.82–2.93)	1.80 (0.97–3.34)
50-<60	19 (17.6)	89 (82.4)	1.26 (0.64–2.50)	1.25 (0.66–2.38)
≥ 60	23 (26.7)	63 (73.3)	1.92 (1.00-3.68)	2.44 (1.18–5.03)*
**Region of country patient comes from**				
Central	46 (26.0)	131 (74.0)	1.00	1.00
Eastern	14 (14.7)	81 (85.3)	0.57 (0.32–0.98)	0.54 (0.28–1.03)
Northern	5 (12.8)	34 (87.2)	0.49 (0.21–1.16)	0.67 (0.21–2.17)
Western	16 (17.4)	76 (82.6)	0.67 (0.40–1.11)	0.68 (0.31–1.51)
**Marital Status**				
Single	6 (30.0)	14 (70.0)	1.00	1.00
Divorced	15 (25.4)	44 (74.6)	0.85 (00.38–1.89)	0.86 (0.40–1.87)
Married	34 (17.0)	166 (83.0)	0.57 (0.27–1.18)	0.59 (0.29–1.18)
Widowed	26 (21.0)	98 (79.0)	0.69 (0.33–1.48)	0.67 (0.31–1.43)
**Residence**				
Rural	40 (17.8)	185 (82.2)	1.00	1.00
Urban	41 (23.0)	137 (77.0)	1.30 (0.88–1.91)	1.05 (0.67–1.64)
**Education**				
None	19 (22.1)	67 (77.9)	1.00	1.00
Primary	27 (18.1)	122 (81.9)	0.82 (0.49–1.39)	0.67 (0.39–1.15)
Secondary	24 (19.2)	101 (80.8)	0.87 (0.51–1.49)	0.75 (0.39–1.42)
Tertiary	11 (25.6)	32 (74.4)	1.16 (0.61–2.21)	1.08 (0.51–2.29)
**Formal employment Status**				
Unemployed	55 (19.2)	232 (80.8)	1.00	1.00
Employed	26 (22.4)	90 (77.6)	1.17 (0.77–1.77)	1.08 (0.67–1.74)
**Prior Information on Cervical cancer**				
No	8 (12.5)	56 (87.5)	1.00	
Yes	73 (21.5)	266 (78.5)	1.72 (0.87–3.40)	1.88 (0.95–3.69)
**Family History of Cervical cancer**				
No/Don’t know	72 (19.4)	300 (80.6)	1.00	1.00
Yes	9 (29.0)	22 (71.0)	1.50 (0.83–2.70)	1.61 (0.88–2.97)
**Level of Referring health facility (HF)**				
Level0(Self-referral)	17 (24.3)	53 (75.7)	1.00	1.00
Private Clinics	3 (12.0)	22 (88.0)	0.49 (0.16–0.37)	0.79 (0.25–2.48)
Level4 (HCIVs)	3 (14.3)	18 (85.7)	0.59 (0.19–1.82)	0.70 (0.22–2.26)
Level5 (DHs)	9 (17.3)	43 (82.7)	0.71 (0.35–1.47)	0.81 (0.38–1.74)
Level6 (RRHs)	20 (14.0)	123 (86.0)	0.58 (0.322–1.03)	0.65 (0.35–1.22)
Level7 (NRHs)	29 (31.5)	63 (68.5)	1.30 (0.78–2.17)	1.36 (0.79–2.35)
**Distance to UCI**				
0-<100 km	34 (23.9)	108 (76.1)	1.00	1.00
100-<200 km	18 (20.9)	68 (79.1)	0.87 (0.53–1.45)	1.50 (0.89–2.55)
200-<300 km	17 (19.1)	72 (80.9)	0.80 (0.48–1.34)	1.98 (0.90–4.36)
≥ 300 km	12 (13.9)	74 (86.1)	0.58 (0.32–1.06)	1.30 (0.55–3.06)
**No of Pre-referral Visits**				
≤ 2 times	22 (25.3)	65 (74.7)	1.00	1.00
> 2times	59 (18.7)	257 (81.3)	0.74 (0.48–1.13)	0.63 (0.41–0.98)*
**Composite stage**				
I/II	52 (21.5)	190 (78.5)	1.00	1.00
III/IV	29 (18.0)	132 (82.0)	0.81 (0.56–1.26)	0.76 (0.50–1.15)
**Duration before seeking care**				
< 3months	32 (39.5)	123 (38.2)	1.00	1.00
3-6months	12 (14.8)	85 (26.4)	0.59 (0.32–1.11)	0.52 (0.29–0.95)*
> 6months	37 (45.7)	114 (35.4)	1.19 (0.78–1.80)	1.19 (0.78–1.82)

UCI = Uganda Cancer Institute

Bold *=Significant at multivariate level with P < 0.05. All factors in the table were adjusted for each other

### Factors associated with disease stage at diagnosis

The study shows that women aged 50 to 59 years were about one and half times more likely to be diagnosed with early stage cervical cancer than those aged less than 40 years, aPR = 1.32 (1.03–1.69). Patients with shorter diagnostic intervals (≤ 3months) were also about one and half times more likely to be diagnosed with early stage cervical cancer, aPR = 1.27 (1.08–1.48). Although married participants were more likely to be diagnosed in early stage, this association was not statistically significant. Patients who first discussed their symptoms with traditional and complementary medicine practitioners or spiritual healers were 35% less likely to be diagnosed with early stage cancer compared to those who discussed their symptoms with a primary healthcare professional, aPR = 0.65 (0.42–0.98) (Table 5).

**Table 5:** Cervical Cancer Stage at diagnosis & associated factors

Factor	Cervical Cancer Stage at diagnosis		Prevalence Ratio (PR)	
	Early stage (I/II) n = 242 (60.1%)	Advanced Stage (III/IV) n = 161 (39.9%)	Unadjusted PR (95% CI)	Adjusted PR (95% CI)
**Age/years**				
< 40	42 (53.2)	37 (46.8)	1.00	1.00
40-<50	82 (63.1)	48 (36.9)	1.19 (0.93–1.52)	1.18 (0.92–1.51)
50-<60	73 (67.6)	35 (32.4)	1.27 (1.00-1.62)	1.32 (1.03–1.69)*
≥ 60	45 (52.3)	41 (47.7)	0.98 (0.73–1.31)	0.97 (0.71–1.32)
**Marital Status**				
Single	12 (60.0)	8 (40.0)	1.00	1.00
Divorced	32 (54.2)	27 (45.8)	0.90 (0.59–1.39)	0.97 (0.63–1.49)
Married	131 (65.5)	69 (34.5)	1.09 (0.75–1.58)	1.18 (0.79–1.76)
Widowed	67 (54.0)	57 (46.0)	0.90 (0.61–1.33)	0.94 (0.63–1.41)
**Residence**				
Rural	132 (58.7)	93 (41.3)	1.00	1.00
Urban	110 (61.8)	68 (38.2)	1.05 (0.90–1.24)	1.07 (0.91–1.27)
**Education**				
None	53 (61.6)	33 (38.4)	1.00	1.00
Primary	88 (59.1)	61 (40.9)	0.96 (0.77–1.19)	0.87 (0.71–1.08)
Secondary	74 (59.2)	51 (40.8)	0.96 (0.77–1.20)	0.83 (0.65–1.05)
Tertiary	27 (62.8)	16 (37.2)	1.02 (0.77–1.35)	0.87 (0.64–1.19)
**Formal employment Status**				
Unemployed	169 (58.9)	118 (41.1)	1.00	1.00
Employed	73 (62.9)	43 (37.1)	1.07 (0.90–1.27)	1.02 (0.84–1.23)
**Prior Information on Cervical cancer**				
No	38 (59.4)	26 (40.6)	1.00	1.00
Yes	204 (60.2)	135 (39.8)	1.01 (0.81–1.26)	0.93 (0.74–1.17)
**Knowledge of PAP Smear**				
No	15 (53.6)	13 (46.4)	1.00	1.00
Yes	227 (60.5)	148 (39.5)	1.13 (0.79–1.61)	1.18 (0.81–1.72)
**Diagnostic interval**				
≤ 3 months	134 (67.0)	66 (33.0)	1.26 (1.07–1.48)*	1.27 (1.08–1.48)*
> 3 months	108 (53.2)	95 (46.8)	1.00	1.00
**Pre-treatment interval**				
≤ 1month	52 (64.2)	29 (35.8)	1.09 (0.90–1.31)	1.11 (0.92–1.34)
> 1 months	190 (59.0)	132 (41.0)	1.00	1.00
**Family History of Cervical cancer**				
No/Don’t know	226 (60.7)	146 (39.3)	1.00	1.00
Yes	16 (51.6)	15 (48.4)	0.85 (0.60–1.21)	0.82 (0.58–1.17)
**Person first discussed symptoms with**				
Health workers	49 (68.1)	23 (31.9)	1.00	1.00
Relatives	179 (60.1)	119 (39.9)	0.88 (0.73–1.06)	0.84 (0.69–1.02)
Spiritual or Traditional healers	14 (42.4)	19 (57.6)	**0.62 (0.41–0.96)**	**0.65 (0.42–0.98)**

Bold* =Significant with P < 0.05. All factors in the table were adjusted for each other.

## Discussion

We found that majority of participants were diagnosed with early stage (stage I&II) cervical cancer. Participants aged 50–59 years and with shorter diagnostic intervals (< 3 months) were more likely to be diagnosed in early stages than those aged less than 40 years and with longer diagnostic intervals. Half of participants were diagnosed early (i.e. within 3 months of first visit to primary healthcare facilities) but less than a third (81/403) of participants were initiated on cancer specific treatments within one month of reporting to the national cancer treatment facility. Participants more likely to be diagnosed early included those referred from district hospitals (Level 5 facility) and with squamous cell carcinoma histology. Participants who were more likely to be diagnosed late included those who first discussed their symptoms with relatives, had > 2 pre-referral visits, and had advanced stage cancer at diagnosis. Older (> 60 years) participants were more likely to initiate cancer specific treatments early (within one month of reporting to the UCI).

In this study, the majority of participants were aged 40–60 years and were diagnosed with early stage (stages I&II) cervical cancer. Our results are similar to findings from Zambia where the median age among 2,121 cervical cancer patients diagnosed between January 2014 and December 2018 was 49 years, and majority of the patients (48%; n = 941) had stage II disease. Stage IV disease was present in only 5.2% (n = 103) of the patients [32]. However, our results differ from most studies in Uganda and other sub Saharan African countries which show that majority of patients with cervical cancer were often diagnosed with advanced stage cancers and experienced poor survival [4, 8, 33–36]. The probable reason for the difference in stage at diagnosis is that our study is a hospital based study that recruited patients that were likely selected by virtue of ability to reach the city hospital unlike the population based studies referred to. Regardless of the aforementioned variations, the early stage at diagnosis in this study is encouraging and may mean that substantial proportion of the population is aware about the need for prompt health-seeking for cervical cancer symptoms. If the trend in early detection persists and is matched with increasing access to quality cancer treatment, then the survival of patients with cervical cancer will improve in the region.

The diagnostic intervals (median 3.1 months) for majority of participants in this study were longer than the desired time interval of less than 3 months. Only half of the participants had diagnostic intervals (time from first visit to primary healthcare facility with symptoms to histology diagnosis of cancer) of less than 3 months. The majority of the patients with long diagnostic intervals had advanced stage cancers at diagnoses. Long diagnostic intervals have been associated with several factors including low awareness of cervical cancer symptoms, preference for help-seeking with traditional and complementary medicine practitioners (T&CMs), low knowledge and acumens of primary healthcare professionals to detect cervical cancer, low education level, and limited access to cancer diagnostic facilities [37–43]. Indeed, we found that patients who first visited traditional and complementary medicine practitioners were more likely to experience longer diagnostic intervals. Participants who first visited spiritual healers and or traditional and complementary medicine practitioners with their symptoms were 23% less likely to be diagnosed with early stage cancer. This finding is similar to results from a study conducted in Botswana among 984 cervical cancer patients diagnosed between January 2015 and March 2020 in which women from rural areas, especially those who first consulted with traditional and complementary medicine practitioners were about twice as likely (OR = 1.61;95%CI: 1.02–2.55) to be diagnosed with advanced stage cancer compared to women from rural areas that had never consulted with the T&CM practitioners [37]. Training the T&CM practitioners on cancer symptoms and the need to promptly refer to biomedical facilities potentially contributes to shorter diagnostic intervals. We also found that most participants first discussed their cervical cancer symptoms with their relatives before visiting the health facilities, and that 53% of the participants who first discussed their symptoms with their relatives were less likely to be diagnosed early (within first 3 months), suggesting the advice they got from their relatives perhaps did not promote prompt health-seeking and diagnosis. Therefore, there is need to increase awareness about cervical cancer symptoms and benefits of prompt health-seeking for symptoms of cervical cancer in the communities. Increasing cervical cancer awareness is likely to contribute towards reducing advanced stage cancer at diagnoses and promote prompt appropriate help-seeking among women especially from the rural communities who are more likely to be diagnosed after longer intervals from symptoms onset. There is evidence from India that creating awareness in the community was associated with significant increase in the proportion of women diagnosed with earlier stage cervical cancer [15].

In this study, participants who visited the primary healthcare facilities more than twice before referral for cancer diagnoses were 25% more likely to be diagnosed late. Similarly, earlier studies have shown that repeated visits to primary healthcare facilities with cervical cancer symptoms is associated with long time to histology diagnosis, advanced stage cancers at diagnosis, and poorer cancer outcomes for cancers including breast and cervical cancers [22, 36, 44–46]. Repeated visits and delayed cancer diagnosis could be due to inability of the primary healthcare professionals to recognize cancer symptoms and initiate diagnostic processes and or refer patients for cancer diagnosis; or due to a lack of the required resources for cancer diagnosis at the primary healthcare facilities. A study in western Kenya showed that a feeling of inadequacy to perform gynaecologic examinations and resource constraints including inadequate facilities to conduct gynaecologic examinations in women presenting with symptoms of abnormal vaginal bleeding and discharge were the major reasons that primary healthcare professionals (nurses/midwives and clinical officers) did not perform gynaecologic tests that could lead to early cervical cancer detection [47]. Therefore, an understanding of the diagnostic processes and challenges at the primary healthcare facilities from perspectives of the primary healthcare professionals could provide the necessary evidence to inform interventions to avoid repeated visits without appropriate diagnosis, shorten time to diagnosis, and hence downstage cervical cancer at diagnosis. One such interventions is training of primary healthcare professionals on symptoms of cancers, and this has been shown to significantly improve knowledge of the healthcare professionals and lead to prompt cervical cancer diagnosis at earlier stages [48]. However, further studies are needed to better understand contextual factors regarding training of the primary healthcare professionals as well as elucidate other interventions to improve the cancer diagnostic processes at the primary healthcare levels. Recent systematic reviews have shown that there are limited studies between 2000 and 2021 on interventions to improve timely cervical cancer diagnosis in the LMICs, and that the few studies that have assessed such interventions used outcomes that have limited clinical relevance to early cancer detection, and have used non-standardized approaches to measurement of study outcomes [49, 50]. Assessing clinically relevant outcomes such as change in stage at diagnosis, diagnostic intervals, and improvement in mortality from cervical cancer require adequate resources with respect to time and finances for follow up; the lack of such resources have perhaps played a major role in limiting such studies in the LMICs, thereby undermining the potential of these countries from coming up with context relevant interventions to improve timely diagnosis of cervical cancers. Increasing funding of interventional studies in the LMICs is a necessary though not sufficient strategy towards improving cancer survival in these countries.

The median pre-treatment interval in this study was 2.4 months. This interval is much shorter compared to findings from a study in Ethiopia involving 242 cervical cancer patients in which the median time to initiation of cancer specific treatment (radiotherapy) was 5.6 months (IQR: 2–9) [8]. In that study, the 5-year overall survival rate (28.4%) was low [8]. The median pre-treatment interval in this study is also shorter than that reported in a study in Botswana where the median pre-treatment interval was 89 days (about 3 months) [41]. Again, the median pre-treatment interval in this study is shorter than the 3 months in a large study in Taiwan involving 9,693 cervical cancer patients diagnosed between 2004 and 2010. In the Taiwan study, majority of patients (96%) received cancer specific treatments within 3 months of histology diagnosis. Patients who received their first cycle of cancer specific treatments after 180 days were more likely to experience poorer survival compared to those who initiated cancer specific treatments within 90 days of diagnoses [12]. The longer the time between diagnosis and initiation of cancer specific treatments, the poorer the survival [12]. We found that older women were more likely to start cervical cancer specific treatments promptly (within 1 month of reporting to the Uganda Cancer Institute) than the younger women (aged < 40 years). We conjecture that the older women could have older biological children who meet the costs of their healthcare, and therefore have increased and accelerated access to cancer staging investigations that are conducted before starting cancer specific treatments. The critical role of family and or spousal support on earlier cancer stage, shortening diagnostic and pre-treatment intervals as well as improved survival from various cancers have been demonstrated among married couples compared to single or divorced partners [51–56]. However, in this study, we have not demonstrated the advantage of being married in respect of cancer stage at diagnosis, and the diagnostic and pre-treatment intervals.

### Study limitations

The interpretations of our findings need to put into considerations possibilities of recall bias since participants retrospectively recalled key dates used to determine the diagnostic and pre-treatment intervals. However, we minimized recall bias by limiting recruitment to patients diagnosed no more than 24 months prior to recruitment and prompting recall through use of key events including Independence Day, and important holidays and religious events such as Christmas day (calendar landmark approach). We also abstracted some of the data from the case notes to validate patients self-reports. Second, the generalization of our findings could be limited by the fact that the study was conducted at a tertiary level facility and included patients selected by their ability to access the city hospital; the patients that did not reach the facility could be uniquely different. However, since the Uganda Cancer Institute is the only national public cancer specialised treatment facility, patients from all over the country seek care there, and therefore the findings from this study can inform national policies and guidelines on cervical cancer early detection and prompt treatment.

## Conclusions

Majority of patients with cervical cancer symptoms delay to receive confirmatory cancer diagnoses. Younger women, patients who first discuss their symptoms with spiritual healers and or traditional and complementary medicine practitioners as well as those who visit the primary healthcare facilities several times before referral for diagnoses are more likely to delay to receive cancer diagnoses and to be in advance cancer stage at diagnosis. There is need for interventional studies that target younger women, traditional and complementary medicine practitioners, and primary healthcare providers in order to generate evidence to inform cancer policies and guidelines to promote prompt health-seeking, early diagnosis for cervical cancer, and prompt initiation of cancer specific treatments.

### Electronic supplementary material

Below is the link to the electronic supplementary material.

Supplementary Material 1

## Data Availability

The data that support the findings of this study are available from the corresponding author upon reasonable request.
