# Supplementary material for: Diagnostic and pre-treatment intervals among patients with cervical cancer attending care at the Uganda Cancer Institute: a cross-sectional study
Source: BMC Womens Health. 2023 Nov 27;23:633. doi: 10.1186/s12905-023-02785-3 (PMC10683271; doi:10.1186/s12905-023-02785-3)
Supplement: Supplementary file 1 — Supplementary Material 1 [file 12905_2023_2785_MOESM1_ESM.docx]

**QUESTIONNAIRE TO DETERMINE THE ASSOCIATED FACTORS ON DIAGNOSIS & PRE-TREATMENT INTERVALS AMONG CERVICAL CANCER PATIENTS AT THE UGANDA CANCER INSTITUTE, KAMPALA-UGANDA**

1. Study Number (Linked with unique patient identifier)…………………………..

**SECTION 1: SOCIO-DEMOGRAPHIC CHARACTERISTICS**

2. What is your date of Birth (How old are you…………­­_Completed years)

…………………………………………………………….

3. Where were you born?

District…………………….County…………………… Sub-county…………………

4. What is your native Language?

………………………………………………………………

5. Where do you reside?

District………………………..

Urban/Town area………………… Rural/Village area………………………

6. What is your Religion?

1. Christian
2. Muslim
3. Traditional religion/Ancestral Worshipper
4. Others Specify)…………………………..

7. What is your marital status?

1. Married c) Nature of Marriage : i) Monogamous ii) Polygamous
2. Unmarried

8. What is the highest level of education/school you have completed?

1. Secondary/Higher level education
2. Primary level
3. No formal education/illiterate

9. What is your husband’s level of education (if married)?

1. Secondary/Higher level education
2. Primary level
3. No formal education/illiterate

10. What is your Current job?

1. Employed (specify)……………..
2. Unemployed
3. Other (specify)……………….

11. What is your husband’s current job (if married)?

1. Employed (specify)……………..
2. Unemployed
3. Other (specify)……………….

12. What is the name of the nearest health facility/hospital from your home?

……………………………………………………………………………………

13. How far is this facility from your home?

……………………………………………………………………………………

14. How far is the U.C.I from your home?

…………………………………………………………………………………..

**SECTION 2: KNOWLEDGE & PERCEPTIONS ON CERVICAL CANCER**

## Risk factors & Symptoms

15. What do you think causes cervical cancer in a woman? Please mention as many causes/risks/things as you can think of (don’t prompt)

………………………………………………………………………………………………

………………………………………………………………………………………………

…………………………………….......................................................................................

………………………………………………………………………………………………

………………………………………………………………………………………………

………………………………………………………………………………………………

16. A list of risks/causes are going to be read out to you. Which of the following do you think are causes of cervical cancer?

|  | YES | NO | NOT SURE |
| --- | --- | --- | --- |
| 1. History of cervical cancer in the family |  |  |  |
| 1. Using contraceptives/FP methods like pill, inject plan, coil |  |  |  |
| 1. Having sexual intercourse at early age |  |  |  |
| 1. Having many children |  |  |  |
| 1. It is sexually transmitted |  |  |  |
| 1. Poor Hygiene |  |  |  |
| 1. Having your menstrual periods at early stage |  |  |  |
| 1. Smoking cigarettes/pipes |  |  |  |
| 1. Poverty |  |  |  |
| 1. Having multiple sexual partners |  |  |  |
| 1. Menopause |  |  |  |
| 1. Witchcraft |  |  |  |
| 1. Viral infection |  |  |  |
| 1. Others stated by Patient (please specify) |  |  |  |
|  |  |  |  |
|  |  |  |  |

17. A list of risks/causes are going to be read out to you. Which of the following do you think are signs of cervical cancer?

|  | YES | NO | NOT SURE |
| --- | --- | --- | --- |
| 1. Abnormal vaginal bleeding (between periods, after sex, postmenopausal) |  |  |  |
| 1. Vaginal discharge |  |  |  |
| 1. Painful Sex |  |  |  |
| 1. Pelvic pain |  |  |  |
| 1. Painful urination (dysuria) |  |  |  |
| 1. Weight Loss |  |  |  |
| 1. Others stated by Patient (please specify) |  |  |  |
| 1. Swelling (tumor) |  |  |  |
|  |  |  |  |

## **SECTION 3: DIAGNOSIS, MANAGEMENT & PREVENTION**

18. Who should get tested for cervical cancer?

1. Any female
2. Married women
3. Unmarried women
4. Sexual workers
5. Other……….

19. Prior to being diagnosed with cervical cancer, had you ever heard about this disease?

1. Yes
2. No

20. If yes, where did you hear this from (source of information)?

1. Newspaper
2. Television
3. Radio
4. Family member/Relative
5. Medical practitioner
6. Friends
7. Other……..

21. Had you heard about Pap smear/Cervical cancer examination?

1. Yes
2. No

22. If yes, what is the purpose of the examination?

1. Screen cervical cancer
2. STI screening
3. Not sure
4. Other………

23. Have you ever undergone a Pap smear examination?

1. Yes
2. No

24. If yes, how often should one get the Pap smear done?

1. Annually
2. 3-5 years
3. Every 5 years
4. Not aware

25. Cervical cancer can be treated by the following methods

1. Surgery
2. Traditional medicine
3. Spiritual intervention/Prayers
4. Radiotherapy/Chemotherapy
5. Not sure
6. Other…………

26. Once detected early, can cervical cancer be prevented?

1. Yes
2. No
3. Not aware

27. Have you heard about the HPV vaccine?

1. Yes
2. No

28. If yes, where did you hear this from (source of information)?

1. Newspaper
2. Television
3. Radio
4. Family member/Relative
5. Medical practitioner
6. Friends
7. Other……………

29. Would you recommend the vaccine being administered to your daughter?

1. Yes
2. No
3. If No, why wouldn’t you recommend this for your daughter?

……………………………………………………………………………

**SECTION 4: PRESENCE OF CERVICAL CANCER RISK FACTORS**

Thank you for sharing the above information. I would like to now ask you a few questions concerning your health care journey until presenting at the U.C.I

|  | **YES** | **NO** | **Don’t Know** |
| --- | --- | --- | --- |
| 30) Do you know any of your family members who has had cervical cancer now or in the past? |  |  |  |
| 31) If yes, who of your family member? | 1. Sister 2. Daughter 3. Mother 4. Aunt 5. Other (Specify)   ………………. |  |  |
| 32) At what age did you start your menstrual periods | ………………………….. |  |  |
| 33) Have your menstrual periods stopped for life (menopause)? |  |  |  |
| 34) Do you have biological children? |  |  |  |
| 35) If yes, how many children do you have? |  |  |  |
|  |  |  |  |
| Are you/Used Contraceptives before? | 1. Yes 2. No |  |  |
| If yes, for how long have you used them? | 1. A year 2. 1-5 years 3. 5-10 years 4. More than 10 years | | |
|  |  |  |  |
| 36) Has a health worker, nurse/doctor ever told you in the past whether you have……… |  | Yes | No |
|  | 1. Cardiac problem |  |  |
|  | 1. HIV/AIDS |  |  |
|  | 1. Diabetes |  |  |
|  | 1. Hypertension/High blood pressure |  |  |
|  | 1. Other Cancer |  |  |
|  | 1. STI |  |  |
|  | 1. Other disease……… |  |  |

**SECTION 5: ASSOCIATED FACTORS ON THE CARE PATHWAY**

37. When did you first notice the signs/symptoms of the cancer?

………/……../………

38. What were the first symptoms you noticed?

1. Pain/bleeding during or after sexual intercourse
2. Discovered during treatment for other disease
3. Fatigue
4. Vaginal discharge
5. Painful urination
6. Others…………………………………………

39. What did you think these signs/symptoms meant?

1. Sexually Transmitted Infection
2. Thought it wasn’t Serious
3. Monthly periods
4. Bewitched/Witchcraft
5. Pregnancy
6. Other……………………………..

40. On noticing these changes, whom did you first discuss them with?

1. Health worker at health facility
2. Relative (Mother/Father/Husband/Other)……..tick/specify which applicable
3. Traditional Healer
4. Spiritual Leader
5. Other…….

41. After you noticed these changes, how long did it take you to visit the health facility/health worker?

1. Immediately to within the first 2 weeks of noticing
2. About a month
3. About 3 months
4. 6 months
5. More than 6 months
6. Other……..

42. What was the reason for deciding to wait that long? (Don’t prompt)

1. I did not think it was serious
2. Busy work schedule
3. No money for transport
4. The health facility was too far
5. Using some treatment
6. Other………………………..

43. Can you kindly tell me how many health providers/units visited in order from the first until coming to the U.C.I? (Number in order of visit)

| **Health provider seen** | **Date** | **Number of times visited** | **What happened/diagnosis made** |
| --- | --- | --- | --- |
| Nearby private clinic |  |  |  |
| Drug store/Pharmacy |  |  |  |
| Traditional healer/herbalist |  |  |  |
| Primary level HF |  |  |  |
| Secondary Health facility |  |  |  |

44. How long did it take for you to eventually visit the U.C.I?

1. Immediately to within the 2 weeks of Referral
2. About a month or two
3. About 3 months
4. 6 months
5. More than 6 months
6. Other……..

45. What was the reason for deciding to take some time?

1. I did not think it was serious
2. Busy work schedule
3. Lack of finances/No money for transport
4. The health facility was too far
5. Using some treatment/herbal medicine
6. Fear of diagnosis outcome
7. Misdiagnosis from early health provider visits
8. Other…

46. From the Time you visited the U.C.I, how long did it take for you to get a diagnosis confirmation?

1. 1 to 2 weeks
2. About a month
3. More than a month……….

47. What was the reason for the time taken?

……………………………………………………………………….

……………………………………………………………………….

48. What treatment have you undergone so far?

1. Surgery
2. Chemotherapy
3. Radiotherapy
4. Other……………

49. How long did it take to start the treatment from the time of diagnosis?

1. 2-3 weeks
2. About a month
3. 2-3 months
4. More than 3 months

50. What was the reason for that time taken?

1. Looking for treatment finances
2. Discussing with family members
3. Opted for traditional treatment
4. Other……………………………………………………

51. Currently how are you able to cater for the medical bills?

1. Using my salary/employed
2. From my business
3. Family/Relatives contributions
4. Husband
5. Other………………………………….

52. In your view, what did you think about the type of treatment recommended on your health?

…………………………………………………………………………………………….

53. In your opinion, do you think the treatment costs at the U.C.I are?

1. Expensive
2. Very Expensive
3. Affordable

54. In your opinion/following this experience, what would you wish the government could do with respect to cervical cancer?

…………………………………………………………………………………………………..

…………………………………………………………………………………………………..

**Thank you for participating. We have come to the end of this interview.**

55. for interviewer: During the interview session, did the participant have any assistance from anyone (family/friend)

1. Yes
2. No

**SECTION 5: TO BE COMPLETED FROM PATIENT’S MEDICAL FILE**

| 56 | Date first seen at the U.C.I | DD/MM/YY |  | |  |
| --- | --- | --- | --- | --- | --- |
| 57 | Date of Referral to U.C.I | DD/MM/YY |  | |  |
| 58 | Name of Referral Health facility |  |  | |  |
| 59 | Date first seen at Referral Health facility |  |  | |  |
| 60 | Date of cervical cancer diagnosis |  |  | |  |
| 61 | Date of first Chemotherapy |  |  | |  |
| 62 | Date of first Radiotherapy |  |  | |  |
| 63 | Staging of Cervical cancer at diagnosis | 1. IA 2. IB 3. 2 4. 3 5. 4 | | | |
| 64 | Cancer Histology | 1. Squamous Cell Carcinoma | | 1. Adenocarcinoma | |

Name of Interviewer………………………………………………………………

Signature of Interviewer…………………………………………………………..

Date of Interview………………………………………………………………….
